# High-Resolution Imaging of Patients with Bietti Crystalline Dystrophy with *CYP4V2* Mutation

**DOI:** 10.1155/2014/283603

**Published:** 2014-09-03

**Authors:** Kiyoko Gocho, Shuhei Kameya, Keiichiro Akeo, Sachiko Kikuchi, Ayumi Usui, Kunihiko Yamaki, Takaaki Hayashi, Hiroshi Tsuneoka, Atsushi Mizota, Hiroshi Takahashi

**Affiliations:** ^1^Department of Ophthalmology, Nippon Medical School Chiba Hokusoh Hospital, 1715 Kamagari, Inzai, Chiba 270-1694, Japan; ^2^Department of Ophthalmology, Juntendo University Urayasu Hospital, 2-1-1 Tomioka, Urayasu 279-0021, Japan; ^3^Department of Ophthalmology, The Jikei University School of Medicine, 3-25-8 Nishi-shimbashi, Minato-ku, Tokyo 105-8461, Japan; ^4^Department of Ophthalmology, Teikyo University School of Medicine, 2-11-1 Kaga, Itabashi-ku, Tokyo 173-8605, Japan; ^5^Department of Ophthalmology, Nippon Medical School, 1-1-5 Sendagi, Bunkyo-ku, Tokyo 113-8602, Japan

## Abstract

The purpose of this study was to determine the retinal morphology of eyes with Bietti crystalline dystrophy (BCD) associated with a *CYP4V2* mutation using high-resolution imaging techniques. Three subjects with BCD underwent detailed ophthalmic examinations. High-resolution fundus images were obtained with an adaptive optics (AO) fundus camera. A common homozygous mutation was detected in the three patients. Funduscopic examination of the three patients revealed the presence of crystalline deposits in the retina, and all of the crystalline deposits were also detected in the infrared (IR) images. The crystals observed in the IR images were seen as bright reflective plaques located on the RPE layer in the SD-OCT images. The clusters of hyperreflective signals in the AO images corresponded to the crystals in the IR images. High-magnification AO images revealed that the clusters of hyperreflective signals consisted of circular spots that are similar to the signals of cone photoreceptors. Most of these circular spots were detected in healthy areas in the FAF images. There is a possibility that circular spots observed by AO are residual cone photoreceptors located over the crystals.

## 1. Introduction

Bietti crystalline dystrophy (BCD) is an autosomal recessive retinal degeneration characterized ophthalmoscopically by many glistening intraretinal dots scattered throughout the posterior pole of the eye. This retinal degeneration was first described in three patients, two of whom were brothers, by Bietti in 1937. He reported the presence of crystalline deposits in the retina and in the stroma of the limbal cornea [1]. In 1968, Bagolini and Ioli-Spada published a follow-up study on the three patients described by Bietti's and also on 6 additional patients with BCD [2]. They reported that BCD was a progressive retinal degeneration with sclerosis of the choroidal vessels ultimately resulting in night blindness and visual field constriction in the third to fourth decade of life.

BCD is a worldwide disease but it is most common in East Asia especially in the Chinese and Japanese populations. It has been estimated to account for 3% of all nonsyndromic retinitis pigmentosa cases and 10% of all autosomal recessive retinitis pigmentosa cases [3, 4].

The gene responsible for BCD,* CYP4V2*, is expressed in the heart, brain, placenta, lung, liver, retina, and retinal pigment epithelium (RPE) in humans [5]. This gene codes for a member of the cytochrome p-450 family whose structure suggests that it may play a role in the metabolism of fatty acids. Biochemical studies showed that patients with BCD have abnormal lipid metabolism, and the biochemical analysis of CYP4V2 showed that it is a fatty acid omega hydroxylase [6, 7]. Histopathology of the retina of a BCD patient showed advanced pan-chorioretinal atrophy with crystals and complex lipid inclusions in the choroidal fibroblasts, corneal keratocytes, conjunctival and skin fibroblasts, and circulating lymphocytes [8]. These findings suggested that BCD may result from a systemic abnormality of the lipid metabolism.

In the early stages of the BCD disease process, the RPE-choriocapillaris complex is affected while the functions of rods and cones are well preserved. With the progression of the atrophy of the RPE-choriocapillaris and choroidal sclerosis, there is an alteration of the electroretinograms (ERGs) and progressive constriction of the visual fields. At the late stage, the best-corrected visual acuity (BCVA) is markedly decreased [9].

At present, the origin and biochemical make-up of the crystalline deposits in BCD patients have not been determined. Several spectral domain optical coherence tomographic (SD-OCT) analyses of the eyes of patients with BCD showed that many hyperreflective spots of varying sizes were present in all layers of the retina in patients with BCD [10–16]. The fundus photographs and SD-OCT images of BCD patients clearly showed that the crystalline deposits were mainly located on the retinal side of the RPE [12].

Adaptive optics (AO) technology has enabled clinicians to view the retina with microscopic lateral resolution [17, 18]. Although this technique has been used to analyze the cone photoreceptor mosaic in eyes with inherited retinal degenerations [19, 20], it has not been used to analyze the eyes of patients with BCD.

Thus, the purpose of this study was to determine the relationship between the retinal morphology and the crystalline deposits. To accomplish this, we studied three patients with BCD by high-resolution imaging including SD-OCT and AO retinal imaging.

## 2. Methods

The protocol of this study conformed to the tenets of the Declaration of Helsinki and was approved by the Institutional Review Board of the Nippon Medical School. A written informed consent was obtained from the three patients after an explanation of the nature and possible complications of the study.

### 2.1. Clinical Examinations

The ophthalmological examinations included measurements of the best-corrected visual acuity (BCVA), determination of the refractive error (spherical equivalent), slit-lamp biomicroscopy, ophthalmoscopy, fundus photography, perimetry, SD-OCT, infrared and fundus autofluorescence imaging, and full-field and multifocal ERGs. The visual fields were obtained by Goldman perimetry and Humphrey Visual Field Analyzer (Model 745i; Carl Zeiss Meditec, Inc., Dublin, California). The Swedish interactive threshold algorithm standard strategy was used with program 30-2 of the Humphrey Visual Field Analyzer. The autofluorescence images were acquired with the TRC-NW8Fplus (TOPCON, Tokyo, Japan). The OCT and infrared images were acquired with a Cirrus HD-OCT (Carl Zeiss Meditec). Full-field scotopic and photopic ERGs were recorded using an extended testing protocol incorporating the International Society for Clinical Electrophysiology of Vision (ISCEV) standards [21]. The mfERGs were recorded using a commercial mfERG system (VERIS Jr. Science; Mayo, Aichi, Japan). This system uses basically the same technology as the Visual Evoked Response Imaging system [22, 23]. The mean luminance of stimulus was 103 cd/m^2^ and the contrast was 95%. The overall stimulus area subtended approximately 20°, and the frame rate was 75 Hz. The pseudorandom stimulus presentation, the m-sequence, was 2^14^ − 1, and each run was divided into eight equal segments with a total recording time of about 4 min.

### 2.2. Genetic Testing

Blood samples were collected from the patients and genomic DNA was isolated from peripheral white blood cells with a blood DNA isolation kit (NucleoSpin Blood XL; Macherey Nagel, Germany). The DNA was used as a template to amplify the* CYP4V2* gene. The coding regions and flanking introns of the* CYP4V2* gene were amplified by polymerase chain reaction (PCR) with published primers [5]. The PCR products were purified (ExoSAP-IT; USB Corp., USA) and both strands of the gene were sequenced with an automated sequencer (Bio Matrix Research; Chiba, JAPAN).

### 2.3. Adaptive Optics Flood Illumination Image Acquisition

Fundus images with microscopic resolution were obtained using the flood-illuminated AO retinal camera (rtx1, Imagine Eyes, Orsay, France) [24]. This system was used in earlier investigations to obtain images of individual cone photoreceptors [18, 20, 25, 26] and other retinal structures [18, 27, 28]. The AO fundus camera illuminates a 4-degree square field with 850 nm infrared flashes to acquire* en face* images of the retina with a transverse optical resolution of 250 line pairs/mm. Successive AO images were taken at adjacent retinal locations with an angular spacing of 2 degrees in the horizontal and vertical directions. This procedure allowed an overlap of at least 2 degrees of the horizontal and vertical images. Prior to each acquisition, the focus depth was adjusted to the region corresponding to the ellipsoid and interdigitation zones (formerly called the inner segment/outer segment (IS/OS) junction and cone outer segment tip (COST) line [29, 30]) in the OCT images. The resulting images were stitched together by superimposing retinal vessel landmarks with an image editing software (Photoshop, Adobe Corporation, Mountain View, CA; GIMP, The GIMP Development Team; Image J, National Institute of Health, Bethesda, MD). The pixel size of the images was typically 0.8 *μ*m when calculated at the retinal plane and the value was adjusted for individual variations in the axial length of the eye [31].

## 3. Results

### 3.1. Clinical Findings

**Figure 1:**
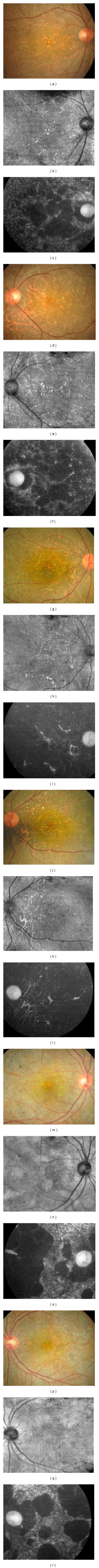
Fundus photographs, infrared images, and fundus autofluorescence images of eyes with Bietti crystalline dystrophy (BCD) with* CYP4V2* mutation. Images from Case  1 ((a)–(f)), Case  2 ((g)–(l)), and Case  3 ((m)–(r)) are shown. Fundus photographs ((a), (d), (g), (j), (m), and (p)), infrared images ((b), (e), (h), (k), (n), and (q)), and fundus autofluorescence images ((c), (f), (i), (l), (o), and (r)) are shown. Fundus photographs of the patients show many small glistening yellowish white spots. Infrared image shows the crystalline deposits clearly. Fundus autofluorescence images show patchy hypofluorescent areas located throughout the degenerative lesion. The crystals observed in the IR images are located only in the areas between the hypofluorescent FAF lesions.

**Figure 2:**
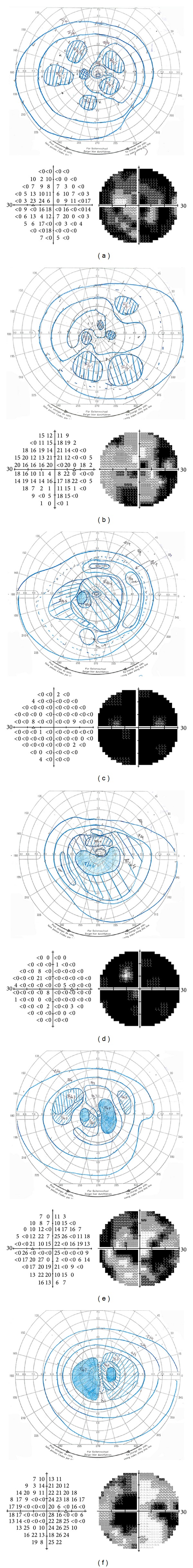
Visual fields of BCD patients. Results of Goldman kinetic perimetry and Humphrey visual field analyzer of Case  1 ((a), (b)), Case  2 ((c), (d)), and Case  3 ((e), (f)). Results from the right eyes ((b), (d), and (f)) and left eyes ((a), (c), and (e)) are shown. Goldmann visual field tests show that the peripheral visual fields are full in all patients. Humphry visual field testing showed a relative central scotoma in all patients.

**Figure 3:**
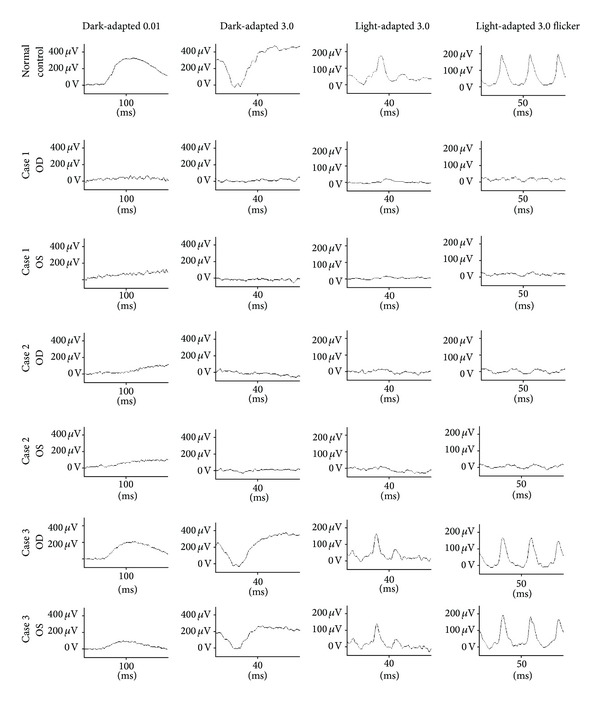
Full-field electroretinograms (ERGs) recorded according to the ISCEV standard protocol in a normal control and BCD patients. Dark-adapted 0.01 (rod), dark-adapted 3.0 (mixed rod-cone), light-adapted 3.0 (cone), and light-adapted 3.0 flicker (30-Hz flicker) ERGs are shown. The rod, mixed rod-cone, cone, and 30-Hz flicker ERGs were nonrecordable in Patients 1 and 2. The rod and the combined rod-cone b-waves of the full-field ERGs were slightly reduced in the left eye of Patient 3. The amplitudes of the b-wave of the cone responses and the amplitude of the flicker responses in Patient 3 were within normal limits.

**Figure 4:**
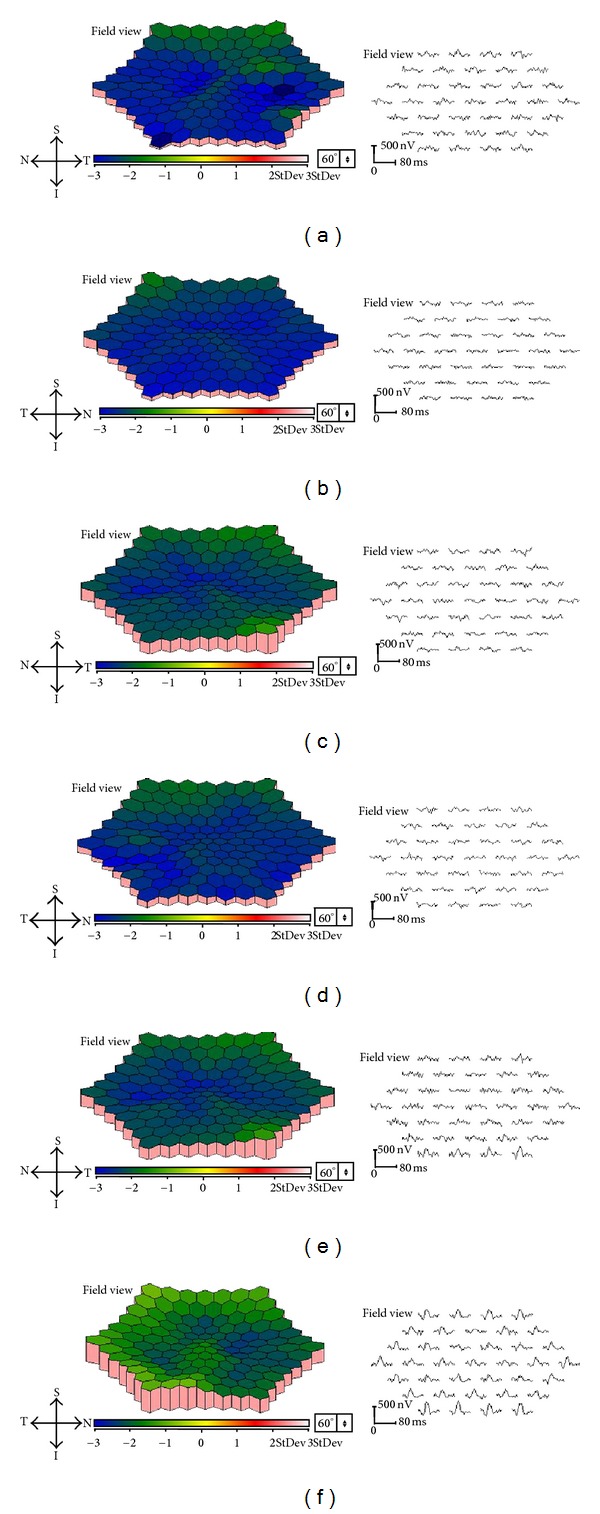
Multifocal ERGs (mfERGs) of BCD patients. Topographic map and local responses of multifocal ERGs recorded from Case  1 ((a), (b)), Case  2 ((c), (d)), and Case  3 ((e), (f)) are shown. Results from right eyes ((a), (c), and (e)) and left eyes ((b), (d), and (f)) are shown. The amplitudes of the mfERGs in the foveal area are severely reduced in all cases.

Patient 1 was a 48-year-old woman who was referred to our department for a differential diagnosis of possible retinitis pigmentosa. She had night blindness since her early forties. Patients 2 and 3 were 42- and 40-year-old sisters. The BCVA of the three patients are shown in Table 1. Slit-lamp examination did not show any fine crystals in the corneal limbus of all patients. However, funduscopic examination showed small, yellowish-white glistening deposits located within the vascular arcades of both eyes of all patients (Figure 1). There was atrophy of the RPE and the central and peripheral retinal vessels were not attenuated. All of the crystalline deposits detected in the fundus photographs were also detected in the infrared images in all patients as reported (Figure 1) [11, 12, 32]. Fundus autofluorescence (FAF) imaging in all patients showed patchy hypofluorescent areas located throughout the lesions. The crystals were observed only in the areas between the hypofluorescent lesions as reported [12]. Goldmann visual field tests showed that the peripheral visual fields (V-4e and I-4e targets) were full in both eyes in all patients (Figure 2). Humphry visual field tests showed a relative central scotoma in both eyes in all patients (Figure 2). The rod, mixed rod-cone, cone, and 30-Hz flicker ERGs were nonrecordable in Patients 1 and 2 (Figure 3). The rod and the combined rod-cone b-waves of the full-field ERGs were mildly reduced in the left eye of Patient 3 (Figure 3). The amplitudes of the b-wave of the cones and the amplitudes of the flicker responses in Patient 3 were within normal limits (Figure 3). The amplitudes of the mfERGs in the foveal area were severely reduced in all patients (Figure 4).

### 3.2. Molecular Genetic Findings

We identified a homozygous mutation, g.IVS6–8_–1delc.802_810delTCATACAGGTCATC-GCT/insGC, in the three patients. This mutation will cause a deletion of the last 8 bp of intron 6 and the first 9 bp of exon 7. An insertion of 2 bp (GC) was also detected. The deletion/insertion mutation is likely to disrupt the 3′-acceptor splice site. The mutation resulted in a mutant transcript so that exon 6 was directly spliced onto exon 8. It has been confirmed that the complete length of 186 bp in exon 7 is skipped resulting in an in-frame deletion mutation, p.V268_E329del [33].

### 3.3. High-Resolution Imaging with SD-OCT and Adaptive Optics Fundus Camera

**Figure 5:**
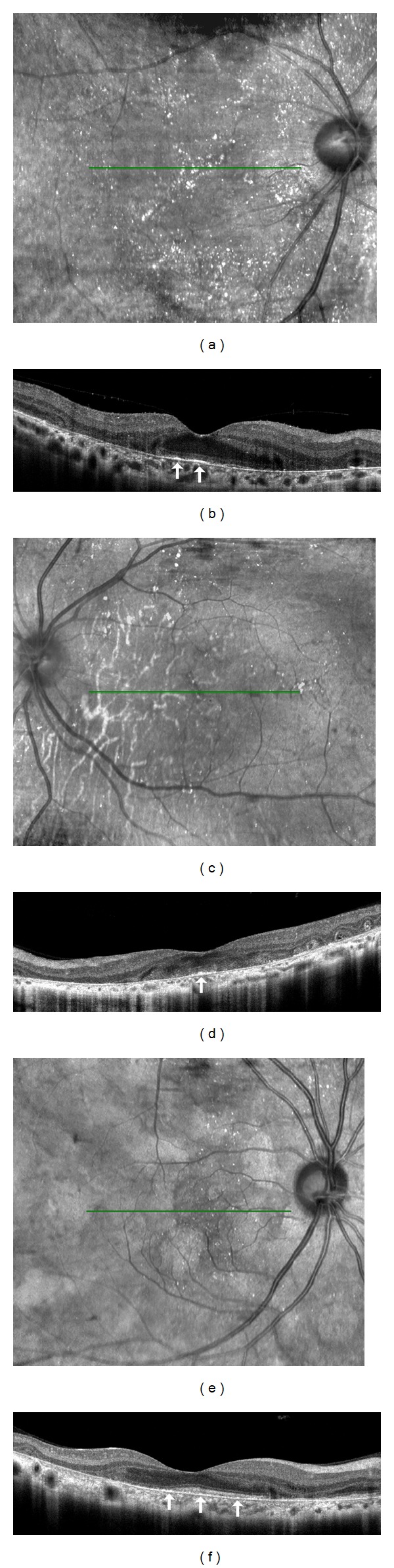
SD-OCT and IR images of BCD patients. IR ((a), (c), and (e)) and SD-OCT ((b), (d), and (f)) images from Case  1 ((a), (b)), Case  2 ((c), (d)), and Case  3 ((e), (f)) are shown. The green horizontal lines in the IR images indicate location of scan line to obtain the SD-OCT images. The RPE and the outer layers of the retina were extensively damaged in all cases. The outer nuclear layer (ONL) of all patients is detectable only in the center of the macular region, but the layer is discontinuous at the peripheral macular region. In Case  2, the ONL is detectable only in the very small region at the fovea. The crystals observed in IR images are seen as brightly reflective plaques located on the RPE layer (arrows). These brightly reflective plaques were found in the areas where the ONL was preserved.

The SD-OCT images of the patients showed many hyperreflective spots of varying sizes in all layers of the retina as reported (Figure 5) [10–16]. The RPE and the outer layers of the retina of the patients were extensively damaged in all cases. The ellipsoid zone was barely visible in Cases  1 and  2 (Figures 5(b) and 5(d)). The ellipsoid zone of Case  3 was still detectable at the nasal macular region; however the zone was discontinuous and blurred (Figure 5(f)). The outer nuclear layer (ONL) of all patients was detectable only in the center of the macular region and the layer was discontinuous at the peripheral macular region. In Case  2, the ONL was detectable only in a very small region at the center of the macula. The crystals observed in the IR images were seen as bright reflective plaques on the RPE layer in the SD-OCT images (Figure 5, arrows). These bright reflective plaques were found only in the areas where the ONL was preserved [16].

**Figure 6:**
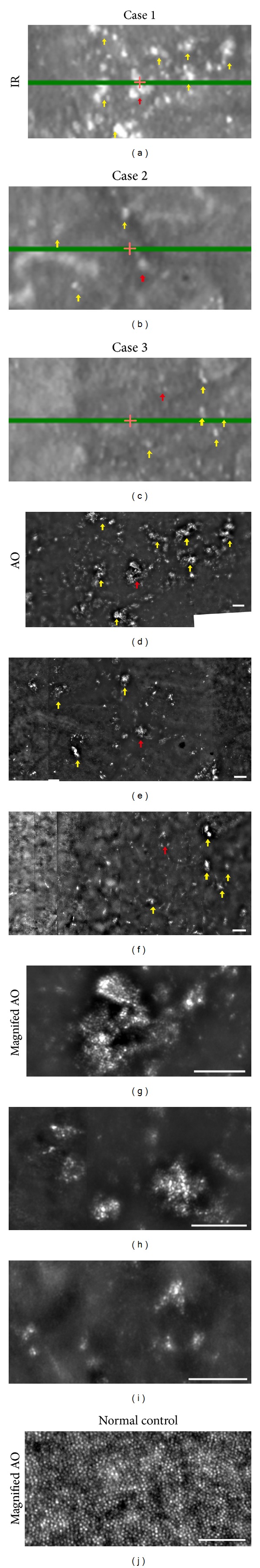
IR and AO images of BCD patients. Magnified IR ((a)–(c)), low ((d)–(f)), and high ((g)–(i)) magnification AO images from Case  1 ((a), (d), and (g)), Case  2 ((b), (e), and (h)), and Case  3 ((c), (f), and (i)) are shown. High-magnification AO images from a normal control are also shown (j). IR images are magnified images of Figures 5(a), 5(c), and 5(e). The red cross in IR images indicates fixation point. The crystals in the IR images are indicated by arrows ((a)–(c)). (a) and (d), (b) and (e), and (c) and (f) are images taken from exactly same region of Case  1, Case  2, and Case  3, respectively. In the low-magnification AO images, the locations of the clusters of hyperreflective signals correspond to the crystals in the IR images ((d)–(f), arrows). High-magnification AO images show that the clusters of hyperreflective signals are cone photoreceptor-like circular spots (Figure 6 red arrows). High-magnification AO image from normal control was obtained from 2-degree nasal of macular center. Bars indicate 100 *μ*m.

The cone mosaic in the AO images was not normal throughout the posterior pole of the eyes (Figures 6(d)–6(f)). In low-magnification AO images, the location of the clusters of hyperreflective signals corresponded with that of the crystals in the IR images (Figures 6(a)–6(f), arrows). High-magnification AO images revealed that the clusters of hyperreflective signals consisted of circular spots that were similar in size to the cone photoreceptors of normal control (Figure 6, red arrows). The spots were of various sizes and the diameter of these circular signals ranged from 4 to 8 *μ*m when they were found within 1 mm of the fovea. Most of these circular spots in Cases  1 and  2 were detected in areas where the ONL was preserved. Unexpectedly, in Case  3 with preservation of the ONL in the SD-OCT images, fewer circular spots were observed than in Cases  1 and  2. Although the low visibility of cone mosaic in Case  3 was consistent with the reduction of the mfERG amplitudes, the blurred ellipsoid zone may have further reduced the visibility of the retinal morphological structures in the AO images.

## 4. Discussion

The biochemical basis of BCD has not been definitively determined, although BCD is most likely related to abnormal oxidation during lipid metabolism because of the mutation of the* CYP4V2* gene [5]. The exact biochemical composition of the crystals in eyes with BCD has not been determined, but histopathological studies have shown that they had morphological characteristics of the crystals. Electron microscopy (EM) confirmed the presence of crystalline materials in circulating lymphocytes and skin fibroblasts in BCD patients [8]. Interestingly, Furusato et al. found lipid-complex inclusion bodies in the melanosomes by EM [34].

The results of several SD-OCT studies suggested that the crystals were mainly located adjacent to the RPE layer [11, 12]. Pennesi and Weleber reported that the brightly reflective plaques were located on top of Bruch membrane and were clearly visible in fundus photographs [11]. Kojima et al. reported that the crystalline deposits observed in patients with BCD were mainly located on the inner side of the RPE layer [12]. Although, the exact location of the crystalline deposits has not been determined definitively, our results suggest that the crystals seen in the fundus photographs and IR images are mainly located on the inner surface of the RPE layer as best seen in the SD-OCT images.

It has been hypothesized that the presence of crystalline deposits in patients with BCD represents the slightly degenerated (i.e., relatively healthy) areas of the retina because the FAF images showed that the crystals were observed only in the areas between the hypofluorescent lesions [12]. Our results also demonstrated that the crystals in the IR images were found adjacent to relatively healthy RPE. In addition, the crystals in the SD-OCT images were detected in areas where the ONL was preserved. These findings suggest that the circular spots found over the crystals in the AO images were located in relatively healthy regions of the retina.

There are several possibilities on why the circular spots are detected in the AO images of BCD patients. The circular spots may be signals from cones, rods, RPEs, or just speckle artifacts. Rods are generally too small to be detected by the flood illuminated AO system (less than 2 *μ*m) and they usually do not exist in the center of macular region. The RPE cells in the AO images are usually detectable only when the photoreceptors are totally degenerated. When the RPE cells are visible in the AO images, they usually appeared as dark reflectance areas outlined by hyperreflective regions. The diameter of RPE cells is larger than 10 *μ*m as determined histologically. Because several images taken from same area by repeated examination reveal almost the same number of circular spots in the same location, they are most likely not speckle artifacts. In some cases of photoreceptor degeneration, a lower cone density in the central macular region makes the diameter of the cones larger [35]. Histopathological examinations have shown that the diameter of the cone photoreceptors is 2 to 4 *μ*m within 0.5 mm of the foveal center [36]. However, they have also been shown to be 7 *μ*m and more in eyes with cone-rod dystrophy [35].

Combining these data, there is a possibility that the circular spots in the AO images in eyes with BCD are residual cone photoreceptors existing over the crystals on the RPE.

With increasing age, the retinal crystals decrease in number or disappear and are replaced by areas of RPE and choroidal atrophy [37–39]. The replacement of the retinal crystals by progressive RPE and retinal atrophy has been confirmed by other studies [1, 2, 40, 41]. Because of these changes, BCD patients eventually develop marked visual impairment usually progressing to blindness by the 5th or 6th decade of life [39] Our results are consistent with these observations because the cone photoreceptors were found mainly over the retinal crystals. Thus, our results indicate that as the crystals become less apparent, the cone photoreceptors are lost in these areas resulting in severe visual impairment.

Our study has a number of limitations. We suggest that the circular spots in the AO images are cone photoreceptors; however we do not have definitive evidence for this. To address this issue, we need to perform histopathological study of BCD eyes or use more advanced imaging device such as AO-OCT. The flood illuminated AO system has better resolution than other imaging devices; however there are still some limitations in obtaining clear images especially when recording images from degenerating retinas. In Case  3, the number of circular spots was fewer than in Cases  1 and  2 despite the relative better preservation of the ellipsoid zone in the SD-OCT images. We suggest that ongoing photoreceptor degenerations could cause swelling of the photoreceptors and make the visibility of the cone photoreceptors worse. The blurred ellipsoid zone may also account for the lower visibility of the retinal morphological structures in Case  3.

In conclusion, we have demonstrated that the circular spots observed by AO are residual cone photoreceptors located over the crystals on the RPE. We could not obtain images of the crystals on the RPE cells and we cannot explain why this was so. Although it was difficult to reveal the nature of crystals, they may have some effect on the survival of the cone photoreceptors. This is because the appearance of circular spots on the crystals looks healthy compared to the blurred AO images on dark places without crystals. However, it is important to note that we have investigated only three patients from two families. The cross-sectional nature of our study did not allow us to draw conclusions regarding the progression of the degeneration of the cone photoreceptors in BCD patients. To address this, systematic longitudinal studies incorporating detailed ophthalmologic assessments in a larger number of cohorts are needed in determining the mechanisms involved in cone degeneration of BCD. The molecular mechanisms causing the degeneration of the cones and formation of the retinal crystals have also not been determined. Further studies are needed and these findings will be helpful in clarifying the pathology of the cone photoreceptor loss in BCD patients and in developing new therapies.

## Figures and Tables

**Table 1:** Summary of the clinical data of patients with ADOA.

Patient ID	Sex	Age	BCVA^a^ (OD/OS)	Refraction OD	Refraction OS	Axial length (mm) (OD/OS)
1	F	48	0.4/0.7	−1.5/−1.75 × 55	−1.25/−1.75 × 115	23.48/23.64
2	F	42	1.0/1.0	−2.5/−1.0 × 90	−0.5/−0.25 × 40	23.91/23.45
3	F	40	0.9/1.0	−0.25/−1.00 × 175	+0.25/−0.50 × 45	22.61/22.10

^a^Best-corrected visual acuity (decimal).
